# Perspective: Vagal nerve stimulation in the treatment of new-onset refractory status epilepticus

**DOI:** 10.3389/fneur.2023.1172898

**Published:** 2023-04-20

**Authors:** Laura Mantoan Ritter, Richard Selway

**Affiliations:** ^1^Epilepsy Centre, Clinical Neurosciences Department, King's College NHS Foundation Trust, London, United Kingdom; ^2^Maurice Wohl Clinical Neuroscience Institute, Institute of Psychiatry, Psychology and Neuroscience, King's College London, London, United Kingdom

**Keywords:** febrile infection-related epilepsy syndrome (FIRES), new onset refractory status epilepticus (NORSE), vagal nerve stimulation (VNS), neuromodulation, refractory status epilepticus (RSE)

## Abstract

**Introduction:**

Resistance to drug therapy is a major hurdle in new-onset refractory status epilepticus (NORSE) treatment and there is urgent need to develop new treatment approaches. Non-drug approaches such as neuromodulation offer significant benefits and should be investigated as new adjunct treatment modalities. An important unanswered question is whether desynchronizing networks by vagal nerve stimulation (VNS) may improve seizure control in NORSE patients.

**Main text:**

We present a summary of published NORSE cases treated with VNS and our own data, discuss possible mechanisms of action, review VNS implantation timing, stimulation setting titration protocols and outcomes. Further, we propose avenues for future research.

**Discussion:**

We advocate for consideration of VNS for NORSE both in early and late stages of the presentation and hypothesize a possible additional benefit from implantation in the acute phase of the disease. This should be pursued in the context of a clinical trial, harmonizing inclusion criteria, accuracy of documentation and treatment protocols. A study planned within our UK-wide NORSE-UK network will answer the question if VNS may confer benefits in aborting unremitting status epilepticus, modulate ictogenesis and reduce long-term chronic seizure burden.

## 1. Introduction

New-onset refractory status epilepticus (NORSE) and its subcategory febrile infection-related epilepsy syndrome (FIRES) are rare, devastating clinical presentations with <500 cases reported in the literature to date (1, 2). They are associated with high case fatality, long-term morbidity and their treatment is not supported by controlled studies (1, 2). Resistance to drug therapy is a major hurdle in managing this group of patients and there is urgent need to develop new treatment approaches. Functional disability is present in up to two thirds of NORSE survivors and subsequent chronic epilepsy is the norm (2–5), but individual reports and our own experience indicates that some people do have good cognitive and functional outcomes, even after prolonged status epilepticus (6). Whether desynchronizing networks by vagal nerve stimulation (VNS) may improve seizure control remains an unanswered question. Long-term studies of VNS in drug-refractory patients have demonstrated a >50% seizure reduction in up to 60% of patients (7, 8) but the evidence base for VNS having the potential to interrupt refractory status epilepticus acutely is low (Class IV) (9). Additionally, whether VNS modulates brain activity directly through electrical stimulation, or also indirectly, via modulation of the immune system, is not completely understood. We first evaluate the current evidence behind the use of VNS in adult and pediatric NORSE cases, then present our own center's experience and propose avenues for future research.

## 2. Key VNS anti-ictal and anti-epileptogenic mechanisms of action

The anti-ictal and anti-epileptogenic mechanisms of action of VNS have been studied extensively in both humans and animal models and comprise stimulation of serotonergic and noradrenergic centers in the brainstem (10), norepinephrine binding in the limbic system (11) and modulation of cortical γ-Aminobutyric acid (GABA) A receptor density (12). These studies however, have not explored the mechanism of action of VNS in models of status epilepticus, and it is unknown whether the same mechanisms are responsible for the acute or subacute interruption of status, as in the long-term reduction of chronic seizures. In this respect, it is interesting to note, that in a human study seizures that were acutely stimulated using VNS had a reduced ictal spread as well as reduced impact on cardiovascular function (13). It is also unknown, whether VNS stimulation early during status epilepticus may prevent the process of epileptigenesis to some extent. It is also conceivable, that the changes in receptor occupancy and density induced by VNS may act synergistically and over the longer-term with concomitantly administered antiseizure medicines (ASM), such as benzodiazepines. More recent developments have seen VNS being used as anti-inflammatory treatment: preclinical evidence suggests that VNS may regulate cytokine expression by upregulating High mobility group box protein 1 (HMGB1) through activation of the cholinergic anti-inflammatory pathway (CAP), a loop formed of ascending vagus afferents, autonomic brain stem, forebrain cortical structures and descending vagus efferents [reviewed in (14)]. Therefore, application of VNS in NORSE patients may provide an immediate and controllable way to modulate ictogenesis and further brain injury due to unremitting seizures and inflammation.

## 3. Reported cases of VNS use in NORSE/FIRES

We searched ClinicalTrials.gov, and PubMed databases using the following search strategy (Supplementary Figure 1 Search criteria): (“VNS” OR “vagal nerve stimulation” OR “vagus nerve stimulation”) AND (“New-onset refractory status epilepticus” OR “NORSE” OR “FIRES” OR “Febrile infection-related epilepsy syndrome”), including cases summarized in previous systematic reviews (9, 15). We reviewed individual case descriptions and excluded patients that did not fulfill the current definitions of NORSE and FIRES (1). Reports included individual case reports and case series; four reports were published as abstracts only (16–18). The amount of detail included in the cases reviewed varied substantially and the description of VNS stimulation parameters and titration protocols was not uniform. Of the 15 cases of NORSE treated with VNS identified (Table 1), 10 were adult (age range 19–49 years) and five pediatric cases (age range 17 months−14 years), nine were male and six female. Eight cases fulfilled criteria for the subtype FIRES, including all five pediatric cases. An etiology was identified in six adult cases [four NMDAR encephalitis (17, 18, 20, 23), one Human Parvovirus B19 infection (25), one AntiGluR encephalitis (22)], eight cases described negative investigation results and could be classified as cryptogenic (cNORSE) (16, 18–21, 24, 25). When documented, patients had tried multiple antiseizure medications (seven cases, average >5) and anesthetic agents (10 cases, average >3) with propofol, ketamine and midazolam the most commonly used anesthetic agents in order of frequency of use. Five patients were started on the ketogenic diet (16, 17, 19), without aborting status epilepticus, although adequacy of ketosis was never documented. In nine cases, a trial of immunosuppression was described, with most patients undergoing a combination regime of pulsed steroids [six cases (6, 16, 18–20, 22)], followed by ivIg [six cases (6, 16, 18–20, 22)], Plasma exchange [four cases (6, 16, 22, 25)] or Rituximab [two cases (19, 25)].

**Table 1:** Patient characteristics, details of VNS implantation and stimulation parameters in published NORSE cases.

**References**	**Age (year)**	**Diagnosis/etiology**	**Duration of NORSE before VNS**	**Day of VNS activation after implantation**	**Day of clinical change after implantation**	**VNS settings at clinical change**	**Seizure outcome**	**Last intervention before seizure reduction**
Bonardi et al. (6)	14	FIRES, cryptogenic	43 days	0	29	OC = 2.25 mA; DC = 16%	Cessation of status	CBD
Luo et al. (19)	1 year 5 months	FIRES, cryptogenic	14 days	15	42	OC = 3mA, PW = 750 μs, ON = 14s, OFF = 1.8 min	Cessation of status	PRP and TPM
Espino et al. (20)	37	FIRES, cryptogenic	30 days	0	7	OC = 1.75 mA, F = 20 Hz, PW = 250 μs, ON = 30 s, OFF = 30 min	Abortion of SE but lifelong epilepsy	Rtiuximab
Espino et al. (20)	33	NORSE, NMDAR encephalitis	9 years	–	–	–	Initial improvement but then no change in seizure frequency	–
Kurukumbi et al. (21)	25	cNORSE	8 days	–	3	–	Seizure free day 3, recurrence of seizures day 6	Magnet swiping 2mA, 60s on
Yamazoe et al. (22)	24	FIRES, anti-GluR encephalitis	14 months	4	10	OC = 0.5mA, PW = 500 μs, ON = 21s, OFF = 3 min, DC = 12%	Seizure free at 2 months, recurrence after 3 months, seizure free at 1 year	None
Alsaadi et al. (23)	46	FIRES NMDAR encephalitis	110 days	0	7	OC = 2.5 mA, ON = 30 s, OFF = 5 min		None
Hoang et al. (16)	40	cNORSE	Unknown	–	–			PRP
Howell et al. (24)	14	cNORSE	14 days	–	–	OC = 1.75mA	–	–
Howell et al. (24)	9.2	cNORSE	–	–	–	–	Seizure reduction by 30%−40%	–
Howell et al. (24)	8.3	cNORSE	–	–	–	–	Seizure reduction by 30%−40%	–
Lin and Ko (17)	19	NORSE, NMDAR encephalitis	Weeks	–	–	–	–	–
Lin and Ko (17)	49	cNORSE	Months	–	–	–	–	–
Shatzmiller et al. (18)	19	NORSE, NMDAR encephalitis	–	–	–	–	–	Cyclophosphamide
Skaff and Labiner (25)	27	NORSE, Human Parvovirus B19 infection	>52 days	–	–	–	Cessation of status	–

CBD, cannabidiol; DC, duty cycle; —, not documented; NORSE, new-onset refractory status epilepticus; FIRES, febrile infection-related epilepsy syndrome; OC, output current; PRP, perampanel; PW, pulse width; TPM, topiramate; VNS, vagal nerve stimulation.

### 3.1. Timing of VNS implantation and titration protocols

VNS was implanted in the acute phase of NORSE in five cases (range 14–30 days from onset), in the chronic phase of treatment-resistant epilepsy (TRE) in seven cases (range 43 days−9 years from onset, Table 2). For the purposes of this article, we defined the acute phase of NORSE, as occurring within the first 30 days of NORSE onset, hypothesizing this to be the phase of acute inflammation and epileptogenesis, based on our experience and the available literature on clinical, electrographic and imaging evolution in NORSE (2–4). Details of VNS parameters and titration paradigms are summarized in Table 2. When documented, VNS was activated either on the same day of implantation or within the first 2 weeks after implantation: output current was rapidly increased (range 0.25–0.75 mA/24 h) to peak amplitudes of 0.5–3 mA achieved over 7–21 days. The most commonly used initial stimulation frequencies were 20–30 Hz, pulse widths of 250–500 μs, the latter later widened to 750 μs. Duty cycle settings started in the “conventional” range (30 s on/3 min off) with increases every 2–7 days. Whilst most cases remained in the conventional cycling range, the fastest cycling documented was 7s on/14s off (24). VNS resulted in a significant clinical change in 10 cases, an average of 16.3 days after implantation when documented (range 3–42 days). Eight reports documented the last drug modification or intervention before status cessation, albeit this was performed long before the 24 h suggested by Redecker et al. (26) as the most appropriate measure for the evaluation of efficacy of an ASM in the treatment of SE: in one case Perampanel was added (16), one case had Perampanel and Topiramate introduced (19), one completed a course of Rituximab on the same day as seizures were aborted and hence may have drawn additional benefit from previous Rituximab treatments (20), one four pulses of cyclophosphamide (18) and one commenced on CBD oil (21), whilst in two cases VNS was the documented last intervention.

**Table 2:** Summary of outcomes in published NORSE cases treated with VNS.

**References**	**Outcome**	**Adverse events due to VNS**	**Last time point of review**	**ongoing numbers of ASM**	**Chronic epilepsy**	**cognitive outcome**	**level of function**
Bonardi et al. (6)	Alive	Coughing and tachycardia, current reduced to 1.75 mA	12 months	Multiple	8 months seizure free	Normal and fluent speech, resumed home schooling	Walking with assistance
Luo et al. (19)	Alive	–	166 days	4 (TPM, VPA, LEV, PER)	Seizure free	Verbal and social behind normal limits	Walk without assistance
Espino et al. (20)	Alive	–	730 days	–	Daily FIAS	Moderate global cognitive and mild emotional involvement, affecting her social life	—-
Espino et al. (20)	Alive	–	2 years after implantation or 11 years after NORSE	–	12 seizures/d	Cognitively impaired	On disability support
Kurukumbi et al. (21)	Death due to comorbidities d17	–		–		
Yamazoe et al. (22)	Alive	–	1 year	–	Seizure free	Severely impaired	Wheelchair assistance
Alsaadi et al. (23)	Alive	–	8 months	0	–	Mild-moderate cognitive impairment	–
Hoang et al. (16)	Alive	–	1 ms after PRP	5	–	–	Not documented, discharge to IP Rehab
Howell et al. (24)	Died d29 in multiorgan failure, no inflammation on postmortem					–	–
Howell et al. (24)	Alive	–	23 years	2–4	–	Mild-mod ID language and memory deficits normal behavior	
Howell et al. (24)	Alive	–	26y	2–4	–	Mod ID language and verbal memory deficits Mild behavior disorder	
Lin and Ko (17)	Alive	–	–	–	–	–	–
Lin and Ko (17)	Alive	–	–	–	–	–	–
Shatzmiller et al. (18)	Alive	–	–	–	–	–	–
Skaff and Labiner (25)	Alive	–	–	–	–	–	–

FIAS, focal impaired awareness seizures; LEV, levetiracetam; PER, perampanel; TPM, topiramate; VNS, vagal nerve stimulation; VPA, valproate.

### 3.2. Outcomes

Cessation of super-refractory status was ascribed to VNS in two cases implanted in the acute (19, 20) and two in the chronic phase (21, 23). Status epilepticus is defined as refractory when it does not respond to first-line benzodiazepines and second-line antiseizure medicines, requiring general anesthesia: if refractory status persists or recurs 24 h or more after general anesthesia or recurs on withdrawing anesthetic medication it is defined as super-refractory (27, 28). Improvement in seizures but then recurrence was documented in two cases (20, 21), no effect in one (24), whilst sustained seizure reduction was documented in three cases: by 30%−40% in two (24), in one enabling weaning of anesthetic agents and leaving the ICU (23). Long-term outcomes were available for 12 cases (summarized in Table 2) and were documented between 1 month−26 years after implantation: two cases implanted in the acute phase died due to multiorgan failure or comorbidities (21, 24). Three patients were documented as seizure-free survivors, seven have ongoing chronic epilepsy (16, 20, 21, 24, 25). Bradycardia as side effect of Vagal Nerve Stimulation was the only adverse event due to VNS documented in one case (24). Functional outcomes were documented in eight survivors: the best cognitive outcome was documented in a 14-year old female (21) who resumed home schooling with normal and fluent speech. In both patients implanted in the acute and chronic phase, cognitive outcomes ranged from at least mild to severe cognitive impairment, whilst the only case described as walking without assistance was implanted in the chronic phase.

## 4. King's college hospital experience

Two adult cases of NORSE were implanted at our center:

Case 1—Late implantation of VNS in TRE phase of NORSE

A 54-year old female was implanted in the chronic phase of NORSE (day 67 from onset) and had failed multiple standard antiseizure medications, anesthetic agents and trials of immunosuppression (steroids, ivIg, Plasma Exchange). Our patient had also undergone an unsuccessful trial of electroconvulsive therapy and repetitive transcranial magnetic stimulation. VNS was switched on immediately after insertion, initial stimulation started with 0.5 mA output current, and gradually increased to 2 mA, 30 Hz, 500 μS, with a duty cycle of 35% (30 s ON and 1.1 min OFF). Case Ictal activity on EEG resolved on day 2 after implantation, allowing gradual tapering of clonazepam and anesthetic agents, and leaving the ICU. She died 46 days after VNS implantation due to an obstructed tracheostomy and cardiac arrest.

Case 2—Early implantation of VNS in acute phase of NORSEin pregnancy

A 30-year old pregnant female in the first Trimester of pregnancy was implanted with VNS in the acute phase (day 26 from onset) of NORSE possibly linked with drug overdose. She had also failed multiple standard antiseizure medications, anesthetic agents and trials of immunosuppression including Anakinra. VNS was switched on the day of implantation with initial output current of 0.25 mA. Output current was further uptitrated to 1 mA in the following 72 h, and increased to 1.25 mA on day 7 post-op. Our patient experienced improvement of myoclonic jerks from day 7 post-implantation and became seizure-free from day 20 post implantation. She regained functional independence during inpatient rehabilitaton, delivered a premature but healthy baby at 33 weeks and has remained seizure free to date (last reviewed 8 months from onset).

## 5. Discussion and perspectives for future research

Studying rare and complex diseases such as NORSE in the real clinical world is challenging, as patients may be subject to multiple and concomitant interventions and the presence of publication bias toward cases with good outcomes is very likely. Due to the paucity of cases and the variable amount of information available within each report, the level of evidence supporting the use of VNS in NORSE is low. Nevertheless, we feel that from the cases summarized in the previous sections and our experience, some general conclusions can be drawn: overall, VNS was a well-tolerated intervention without significant adverse effects in the short or long term, both in cases implanted acutely or in the TRE phase, supporting its safety even in pregnancy. Whilst it is not possible to determine a stimulation threshold effect leading to seizure cessation, most patients had VNS switched on either immediately or within the first few weeks of implantation at conventional—not high frequency—cycling rates and the output current increased over a short period of time (days to weeks). In the three cases, including ours (22, 23), where VNS was the last intervention before seizure cessation, clinical changes occurred within 7–10 days of implantation and benefit was sustained long term, in keeping with a recent meta-analysis of the effect of VNS in refractory status epilepticus (9). Beneficial effects reported include not only cessation of status but also the ability to wean anesthesia and assess patients's level of consciousness and neurological status. These positive effects were reported when VNS is implanted both in the acute and TRE phase. Since most if not all NORSE survivors go on to develop chronic epilepsy (2, 4, 5), we suggest that implanting VNS in NORSE should always be considered for its chronic neuromodulatory effect to reduce seizure burden in the long term and may also aid reducing ASM burden. Whether earlier implantation allows earlier control of status by acutely desynchronizing ictal rhythms and limiting seizure spread, and whether it would lead to better functional outcomes is unknown and should be put to the test in future trials. At King's College Hospital, a Charles Sykes Memorial Grant is supporting the set up of a multi-center “N-of-1 trial” series to study the efficacy and mechanism of action of VNS in the treatment of NORSE. N-of-1 trials are considered to be among the most relevant and rigorous study designs for assessing individual patent's treatment efficacy in rare diseases, such as NORSE, where a conventional randomized trial design would not be feasible. We will embed this study into a UK-wide NORSE network (NORSE-UK), including all major tertiary neuroscience centers in the UK, and would welcome international collaborators. Our research will develop electrophysiological and serological biomarkers to predict and monitor response to VNS in NORSE, and may become relevant for the treatment of other drug resistant forms of SE.

## Data availability statement

The original contributions presented in the study are included in the article/Supplementary material, further inquiries can be directed to the corresponding author.

## Author contributions

LMR and RS designed the work. LMR wrote the manuscript. Both authors revised the manuscript, read and approved the final version.
