# Supplementary figures and images for: Perspective: Vagal nerve stimulation in the treatment of new-onset refractory status epilepticus
Source: Front Neurol. 2023 Apr 20;14:1172898. doi: 10.3389/fneur.2023.1172898 (PMC10157031; doi:10.3389/fneur.2023.1172898)

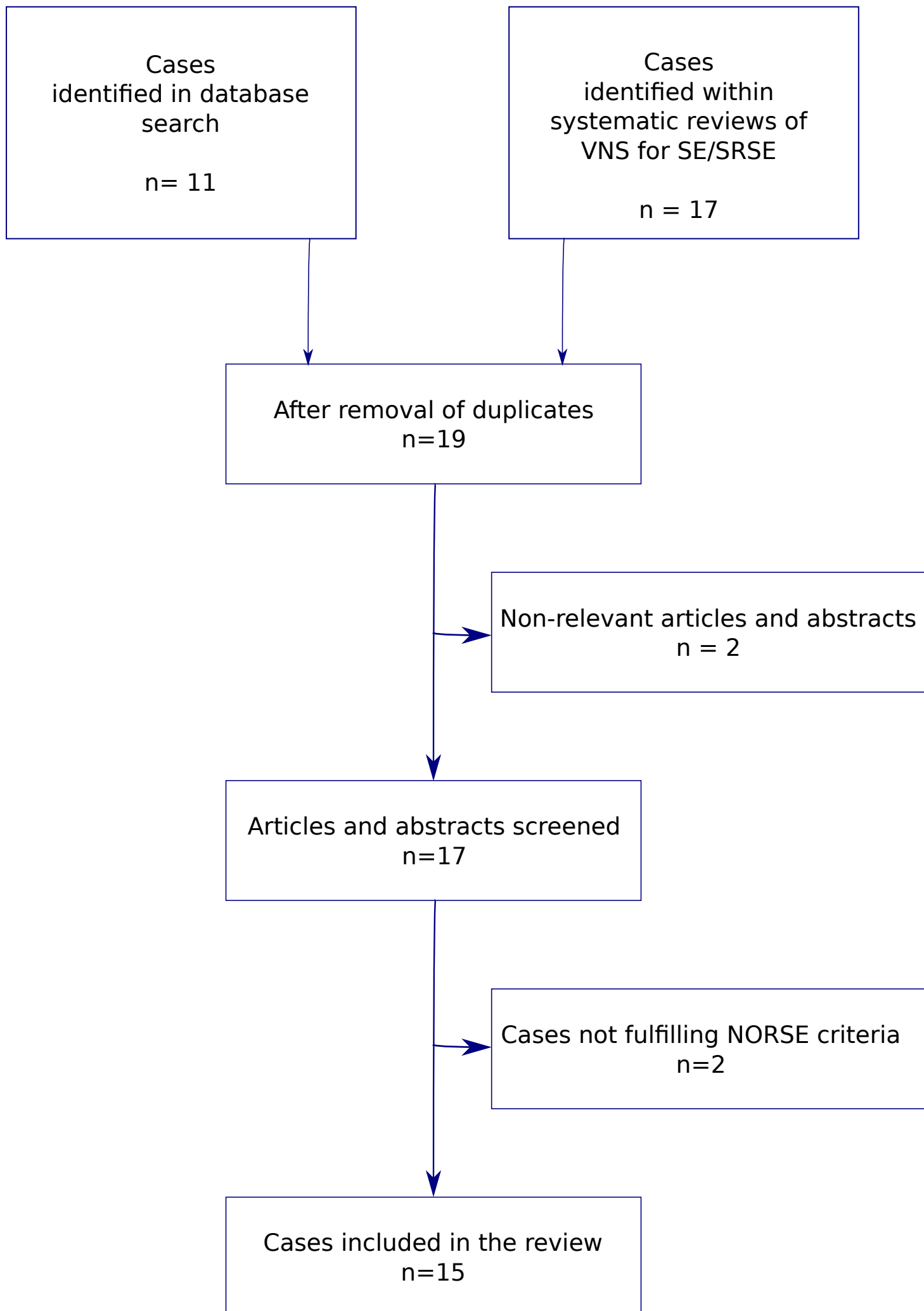

Supplement: Supplementary file 1 [file Data_Sheet_1.PDF]
